# Vascular Calcification Heterogeneity from Bench to Bedside: Implications for Manifestations, Pathogenesis, and Treatment Considerations

**DOI:** 10.14336/AD.2024.0289

**Published:** 2024-04-20

**Authors:** Kuo-Cheng Lu, Kuo-Chin Hung, Min-Tser Liao, Li-Jane Shih, Chia-Ter Chao

**Affiliations:** ^1^Division of Nephrology, Department of Internal Medicine, Taipei Tzu Chi Hospital, Buddhist Tzu Chi Medical Foundation, New Taipei, Taiwan.; ^2^Division of Nephrology, Department of Internal Medicine, Fu Jen Catholic University Hospital, School of Medicine, Fu Jen Catholic University, New Taipei, Taiwan.; ^3^Division of Nephrology, Department of Internal Medicine, Min-Sheng General Hospital, Taoyuan, Taiwan.; ^4^Department of Pharmacy, Tajen University, Pingtung, Taiwan.; ^5^Department of Pediatrics, Taoyuan Armed Forces General Hospital, Hsinchu Branch, Hsinchu, Taiwan.; ^6^Department of Pediatrics, Tri-Service General Hospital, National Defense Medical Center, Taipei, Taiwan.; ^7^Department of Medical Laboratory, Taoyuan Armed Forces General Hospital, Taoyuan, Taiwan.; ^8^Graduate Institute of Medical Science, National Defense Medical Center, Taipei, Taiwan.; ^9^Division of Nephrology, Department of Internal Medicine, National Taiwan University Hospital, Taipei, Taiwan.; ^10^Division of Nephrology, Department of Internal Medicine, National Taiwan University College of Medicine, Taipei, Taiwan.; ^11^Graduate Institute of Toxicology, National Taiwan University College of Medicine, Taipei, Taiwan.; ^12^Center of Faculty Development, National Taiwan University College of Medicine, Taipei, Taiwan

**Keywords:** aortic calcification, atherosclerosis, heterogeneity, mineral bone disorder, radiomics, vascular calcification

## Abstract

Vascular calcification (VC) is the ectopic deposition of calcium-containing apatite within vascular walls, exhibiting a high prevalence in older adults, and those with diabetes or chronic kidney disease. VC is a subclinical cardiovascular risk trait that increases mortality and functional deterioration. However, effective treatments for VC remain largely unavailable despite multiple attempts. Part of this therapeutic nihilism results from the failure to appreciate the diversity of VC as a pathological complex, with unforeseeable variations in morphology, risk associates, and anatomical and molecular pathogenesis, affecting clinical management strategies. VC should not be considered a homogeneous pathology because accumulating evidence refutes its conceptual and content uniformity. Here, we summarize the pathophysiological sources of VC heterogeneity from the intersecting pathways and networks of cellular, subcellular, and molecular crosstalk. Part of these pathological connections are synergistic or mutually antagonistic. We then introduce clinical implications related to the VC heterogeneity concept. Even within the same individual, a specific artery may exhibit the strongest tendency for calcification compared with other arteries. The prognostic value of VC may only be detectable with a detailed characterization of calcification morphology and features. VC heterogeneity is also evident, as VC risk factors vary between different arterial segments and layers. Therefore, diagnostic and screening strategies for VC may be improved based on VC heterogeneity, including the use of radiomics. Finally, pursuing a homogeneous treatment strategy is discouraged and we suggest a more rational approach by diversifying the treatment spectrum. This may greatly benefit subsequent efforts to identify effective VC therapeutics.

## 1. Introduction: vascular calcification as a millennium-old pathology

Vascular calcification (VC) refers to the deposition of ectopic calcium alone or calcium-containing mixed crystals within vascular walls that are visible pathologically and/or radiologically. VC was first reported in ancient Egypt [1]. In the Horus study, investigators used whole-body computed tomography to screen mummies for atherosclerosis based on calcified plaques, and showed that 34%, 20%, and 4% of individuals had arterial calcification, aortic calcification, and coronary artery calcifications, respectively [2]. During the ancient period, the presence of aortic calcification mainly affected the older population, presumably those with better survival, which gives rise to the hypothesis that VC represents a persistent pro-calcific tendency to protect youngsters from premature death related to fractures while becoming harmful later in life [3]. It was not until the advancement of modern medicine and technology that we understood the contradictory but closely related pathophysiological processes between bone remodeling and VC [4].

Based on the speculation that VC is common in older adults, it is perceived as a marker of chronologic aging. In the Framingham study, VC in the form of aortic calcification was detectable in 7-15% of patients younger than 45 years, but the number increased to nearly 100% in those over 75 years of age [5]. Findings from the Multi-Ethnic Study of Atherosclerosis also showed that thoracic and abdominal aortic calcifications occurred in 35% and 73% of participants, respectively [6]. Similar estimates were obtained by our group [7]. Currently, VC is perceived as a subclinical cardiovascular risk factor, exhibiting a strong linkage to traditional risk traits such as advanced age, the presence of diabetes mellitus, hypertension, greater body mass index, and smoking [8]. A meta-analysis consistently demonstrated that abdominal aortic calcification was associated with 81%, 64%, and 72% higher risk of coronary events, cardiovascular events, and cardiovascular mortality, respectively, in the general population [9]. These prognostic influences become more prominent in specific at-risk populations, including those with chronic kidney disease (CKD) and end-stage kidney disease (ESKD), and another meta-analysis revealed that greater coronary calcification was associated with a 2- to 3-fold higher risk of cardiovascular events and mortality among those with CKD [10]. VC has been touted as the vascular manifestation of CKD-mineral bone disorder (CKD-MBD), which encompasses divalent ion imbalances, osteodystrophy and ectopic calcification [11, 12]. Besides its prognostic influences, VC contributes to increased vascular stiffness, soaring pulse wave velocity, precipitating myocardial remodeling, and diastolic dysfunction [13]. We previously showed that VC acts synergistically with myocardial hypertrophy and ventricular geometry regarding its prognostic influence [14, 15]. Finally, VC can directly increase the probability of physical deterioration in older adults [16], which is another important reason that close attention should be paid to this adverse pathology. A newer subtype of frailty, vascular frailty, has been proposed recently to illustrate the accelerated aging process attributable to VC [17].

## 2. VC heterogeneity: a conceptual outline

Despite a wealth of knowledge about the pathological importance of VC, effective treatments remain out of reach [18]. Novel candidates are being tested experimentally and/or clinically [19, 20]; however, none have successfully entered the clinical realm. Part of this therapeutic nihilism may result from the failure to appreciate the diversity of VC regarding its clinical presentations, risk associates, and anatomical and molecular pathogenesis, which may impact clinical management strategies. Indeed, previous literature frequently grouped VC as a single pathological entity [21], but accumulating evidence refutes its conceptual and content uniformity. Prior observations in the CKD population showed that VC was not a single disease entity and CKD-associated VC should be subdivided further based on distinct features [22, 23]. Therefore, we used the term “heterogeneity” as an intuitive approach to describe variations in the aforementioned aspects related to VC. This terminology has also been adopted by others recently [24]; however, they focused primarily on VC in the CKD population, with little mention of evidence in other at-risk populations. Therefore, in this review, we summarize multiple data dimensions to support the notion of VC heterogeneity and its clinical implications.

### 2.1 Through the lens of VC morphological diversity

The heterogeneity of VC can be easily appreciated from the arterial segments involved and inter-individual variations in severity. Studies have revealed that the prevalence of arterial calcification varies depending on the arterial segments, and a small-scale study showed that in the same group of older patients, carotid and coronary calcifications were present in 53.4% and nearly 100% of participants, respectively [25]. The distribution of calcification severity also tended to be more severe for coronary calcification, whose calcification scores could reach 10-fold higher than those for carotid calcification [25]. Razavi *et al*. showed that at least one-third of older adults with severe aortic valve calcification, another form of cardiovascular calcification, did not have coronary artery calcification [26]. Even in the same individual, the pace of VC progression differs among sites. A computed tomography-based study revealed that most participants had only one arterial segment with severe VC progression, whereas others did not [27]. According to a population-based study, the rate of VC progression over the years varied from 33.4% for valvular calcification to 69.6% for coronary artery calcification and 72.4% for abdominal aortic calcification [28]. Interestingly, the degree of cardiovascular risk elevation associated with the presence of VC can be influenced by other clinical features and can predict future cardiovascular risk [29].

However, the absence of VC in certain at-risk populations and its related features also supports the notion that VC is a heterogeneous process, as some patients may be immune to this pathology despite being at risk. Razavi *et al*. reported that younger age, lower total cholesterol-to-high-density lipoprotein cholesterol ratio, and lower phosphate levels were associated with a higher probability of low or absent coronary calcification [26]. Results from patients with hypertension in the Multi-Ethnic Study of Atherosclerosis also indicated that age, waist circumference, fasting glucose, and cholesterol levels significantly affected the probability of VC absence over 10 years [30]. These findings undoubtedly inspire more in-depth investigation into the variable nature of VC initiation.

Based on the observed morphological diversity of VC, it would be tempting to dissect this concept of VC heterogeneity from a pathophysiological perspective. In the following sections, we elaborate on VC heterogeneity based on macroscopic (tissue pathology) and microscopic (molecular pathogenesis) phenotypes. A more precise understanding of these differences is expected to assist in the interpretation of differences in VC etiology, presentation, risk factors, and prognostic influences.

## 3 VC heterogeneity: potential origins

**Figure 1.**
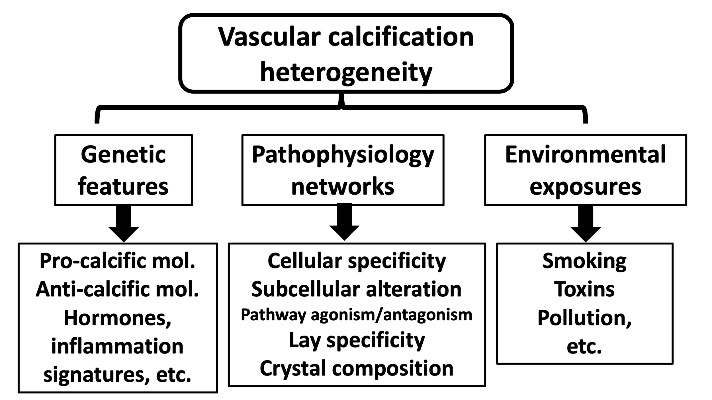
Outlining the origin of vascular calcification heterogeneity.

The origin of VC heterogeneity likely stems from a combination of genetic features that determine pathophysiology and environmental exposure (Fig. 1). A systematic review summarized that biological sex has important effects on the risk of VC [31], and Wu *et al*. speculated that biological sex-related differences in VC probability could result from differential expression or levels of vitamin D, anti-calcific molecules (matrix Gla protein), soluble klotho, and other hormones (parathyroid hormone, fibroblast growth factor-23, and sclerostin). Noxious environmental stimuli, such as active and passive smoking [32], cadmium exposure [33], and air pollution [34] have also been suggested as modifiers of the probability of VC worsening. Dietary fiber content has also been found to modulate the risk of VC development and worsening among those with CKD [35]. However, if we look deeper, differences in vascular tissue susceptibility and alterations in molecular interplay underlie the scene. Pathophysiological differences may account for the majority of observable VC heterogeneities. Therefore, we further elaborate on the cell and tissue specificity of the origin of VC heterogeneity in the ensuing sections.

### 3.1 Pathogenetic heterogeneity: cell specificity and subcellular and molecular variations

Observations made centuries ago postulated that VC originated from a generalized calcifying tendency manifesting in wrong tissues [1] and that all tissues exhibit such a tendency for repair purposes. VC is also attributed to extraosseous calcification related to dystrophic degeneration after injury [36]. It was subsequently discovered that VC is more of a downstream event performed by culprit cells, assuming an active osteoblastic phenotype with osteoid-like matrix secretion [37]. Cells participating in osteoblastic transdifferentiation are mostly vascular smooth muscle cells (VSMCs), although some postulate that endothelial-to-mesenchymal transition, macrophages, or circulatory infiltrating cells provide other sources of osteoblast-like progenitor cells [38]. Furthermore, macrophages and endothelial cells can indirectly contribute to VC, depending on epigenetic regulators [39]. It is plausible that the cellular origin of osteoid matrix deposition in vascular tissues, be it a single cell type or different combinations of calcifying cell species, may play a role in determining VC heterogeneity. This is indirectly supported by pathological results showing that VSMCs and macrophages were each in close proximity to sheet-like and nodular calcifications in vascular tissues, respectively [40].

VSMCs exposed to osteogenic media exhibit prominent upregulation of osteoblast or osteoprogenitor genes, including *Runx2/Cbfa1*, *Osterix*, osteoprotegerin, osteopontin, alkaline phosphatase, and *COL1A1* [41, 42]. Although the culprit VSMCs exhibit an osteoprogenitor and/or osteoblastic signature, significant transcriptomic differences remain between these transdifferentiated cells and mature/committed osteoblasts. The expression levels of osteoblast markers, including RUNX2, osterix, and osteopontin, are 40-fold lower in calcifying VSMCs than in mature osteoblasts [43]. Furthermore, alkaline phosphatase activity is 100-fold lower in calcified VSMCs [43]. From this perspective, under procalcific stimuli, VSMCs may migrate along the differentiation spectrum between myogenic and osteoblastic lineages. Thus, the degree of proximity to the full osteoblast phenotype influences the degree of calcification and VC heterogeneity. Indeed, bone morphogenetic proteins have been shown to postpone myogenic differentiation while promoting osteogenic differentiation in myoblasts [44], and similar regulatory pathways have been observed in VSMCs [45]. These findings suggest that differentiation uncertainty is another reason for VC heterogeneity.

As we delve in further, calcifying pathway heterogeneity becomes more important. Using human arterial specimens from different segments (thoracic and abdominal aortas, carotid, femoral, and infrapopliteal arteries), Espitia *et al*. reported that the femoral arteries had the most severe calcifications associated with distinct transcriptomic patterns from their constituent VSMCs [40]. Most severely calcified arteries have significant upregulation of cellular carbohydrate metabolism and transforming growth factor-β (TGFβ) signaling, while downregulating immune response genes, compared to other segments. Existing literature summarized that multiple agonistic signaling interact during the course of VC initiation and propagation, including bone morphogenetic proteins, Wnt/β-catenin, TNFα, SIRT, other antioxidant/proinflammatory pathways, and TGFβ [46]. The degrees of tissue senescence, systemic inflammation, and endogenous metabolites may differentially affect each pathway and modulate calcification [47]. Fluctuations in the agonistic and antagonistic activities of pathogenic VC signaling serve as an alternative impetus for inducing VC heterogeneity. In addition, our group recently discovered that pro-calcific stimuli may be mutually antagonistic; using *in vitro* and *ex vivo* models, we demonstrated that exogenous TGFβ administration intensified VC severity, whereas other pro-calcific factors triggered a downregulation of endogenous TGFβ expressions [48]. These findings further imply that the primary drivers of VC determine the predominant pathway(s) leading to calcification, and that VC subtyping, as well as the selection of individual druggable targets, can be a potential therapeutic option. Similar pathophysiologic diversity is observed with regard to valvular calcification process, which involves pro-inflammatory, pro-fibrotic, and pro-osteogenic stimuli, oxidative stress, accompanied by metabotoxic exposure of valvular interstitial cells [49]. Interestingly, in the Multiethnic Study of Atherosclerosis study, fibroblast growth factor-21 levels exhibited strong correlation with VC worsening but not valvular calcification progression [50], lending support to the heterogeneity even between these two types of ectopic calcification. We previously also discovered that aortic valve calcification interacted with VC regarding cardiovascular risk prediction in ESKD patients and had its influences independent of VC [51].

Scales as small as the subcellular fractions also account for VC heterogeneity. Calcified vessels, particularly transdifferentiated VSMCs, release calcification-inducing particles or exosomes that exert paracrine or autocrine effects leading to local propagation. These small vesicles contain tetraspanins, proteins related to calcium-binding and extracellular matrix, cytokines/chemokines, and microRNAs. The process of vesicular secretion appears to be highly regulated [52]. Calcification-inducing particles isolated from human calcified aortae have significantly diverse sizes and shapes and contain a high percentage of calcium, phosphorus, and oxygen relative to particles from normal vessels [53]. It remains unclear whether larger particles carry different cargos or exert different levels of calcification modulation. However, the content of specific miRNAs carried by VSMC-released particles can play an important role. For example, the presence of anti-calcific miRNAs, such as miR-125b and miR-378a-3p, in circulating particles is inversely correlated with the risk of VC [54-56]. A literature review comprehensively summarized the functional roles of multiple positively and negatively affecting miRNAs in VC propensity [57], and some of these identified miRNAs exhibit pleiotropic vasculo-active effects [58]. Different degrees of “calcium stress,” resulting from combinations of various proinflammatory cytokines and growth factors, can alter the composition of secreted particle cargo and act as a nidus for crystal formation [59]. As the machinery responsible for regulating the speed, amount, and constituents of calcification-inducing particles remains unclear, VC heterogeneity may emerge. This is supported by the findings of Kirsch *et al.*, who described that inflammation severity might modulate the degree of VC within the aortic segments and a predilection for involvement of the abdominal aorta instead of the thoracic aorta [60]. A broader perspective can also come from speculations from different interactomes shown in VC phenotypes with different origins [61].

### 3.2 Pathogenetic heterogeneity: layer specificity and composition

It is well established that in vascular tissues, tunica intima and media can calcify separately or jointly, each representative of atherosclerotic and metabolic calcification, respectively [62]. Tunica intima calcification tends to occur late during the progression of vasculopathy and is strongly associated with traditional cardiovascular risk factors and inflammation. In contrast, medial calcification tends to emerge early during metabolic stress and has a stronger relation with divalent ion imbalance, metabolic stress, and impairment of anti-calcific defenses [63]. Intimal calcification leads to luminal stenosis and flow obstruction, accompanied by end-organ ischemia, whereas medial calcification culminates in wall stiffening and increased peripheral resistance. Despite the differences in etiological and clinical sequelae, the identification of VC according to histopathological layers is not always easy. This is further complicated by the fact that other VC culprit cells, such as macrophages, can induce paracrine effects by stimulating the release of exosomes to regulate calcification progression [64]. These effects can promote VC pathogenic synergism across the intimal and medial layers, obscuring the accurate identification of the calcification layers.

The physicochemical properties of calcified vascular tissues are another source of VC heterogeneity. Using scanning X-ray diffraction analyses, researchers have shown that arterial microcalcifications differ in crystal composition between those with and without CKD. Those with CKD have more calcium phosphate apatite at the core of the calcification, whereas those without CKD were more likely to have whitlockite [65]. Ultrastructural changes related to local Mg actions may arise from different pathophysiology and are not discernible by simple calcification staining alone. Therefore, discrepancies in tissue calcification composition constitute another origin of VC heterogeneity.

## 4. Implications for clinical practice

The heterogeneity of VC has important clinical implications. The understanding that VC has diverse etiologies manifesting as a wide array of subtypes resulting from complex molecular pathologies and clinical feature variations points to the need for individualization of diagnosis and management approaches. The following sections describe the effects of VC heterogeneity on the clinical management of patients with VC.

### 4.1 Clinical manifestations and risk correlates based on the concept “VC heterogeneity”

Calcification is a heterogeneous and dynamic process that involves vascular tissues. VC heterogeneity manifests in a wide range of presentations. The distribution of calcium crystals in atherosclerotic plaque calcifications is uneven and varies according to plaque size, arterial morphology (bifurcation versus trunk), and fibrous layer thickness [66]. Even within the same individual, specific arteries (such as femoral arteries) may exhibit the strongest tendency for calcification, and the calcified parts are denser and nodular/sheet-like [40]. Despite this calcification heterogeneity, the prognostic value of VC may only be detectable if a detailed characterization of calcification morphology and features is performed. Indeed, the mere feature of plaque calcification has been reported to have little association with the risk of ipsilateral stroke [67], whereas other plaque features exhibit a better correlation. However, computed tomography-identified mean and peak coronary calcium density as a more precise heterogeneity estimation can improve outcome predictability in the general population, in addition to the traditional cardiovascular risk prediction algorithm [68]. Plaque calcification with nodular morphology is also a surrogate measure of calcification heterogeneity and is associated with coronary stenting failure and stent under-expansion [69]. Similar associations have been observed in patients with carotid plaques who underwent endarterectomy [70]. The severity of intimal calcification decreases from the proximal to the distal arterial segments, whereas that of medial calcification increases from the proximal to distal segments [71]. This heterogeneity regarding coronary plaque calcification is also supported by paradoxical findings illustrated below; the presence of coronary plaque calcification indicates a higher burden of atherosclerotic coronary disease and increases cardiovascular risk. However, non-calcified coronary plaques still cause luminal stenosis [72], and the degree of coronary plaque calcification was found to be protective of future coronary atherosclerosis progression [73].

VC heterogeneity is also evident in that the risk factors for VC vary among different arterial segments and calcification pathological layers. Human data showed that different arterial segments reacted differently with regard to their calcification susceptibility to air pollution, and a meta-analysis showed that fine particulate matter exposure correlated more closely with carotid arteriopathy than with coronary calcification [74]. The degree of coronary calcification was significantly associated with carotid calcification, but with side deviations (left > right) [25]. Interestingly, hypertension correlated with higher carotid calcification scores, whereas diabetes and dyslipidemia correlated with higher coronary calcification scores. In addition, Vos *et al*. reported that smoking history and hypertension significantly increase the risk of predominantly intimal calcification, whereas diabetes mellitus and previous vascular disease increased the risk of medial calcification [75]. This “manifestation heterogeneity” can also be observed using artificial intelligence-assisted extraction of quantitative and reproducible metrics, called radiomic features, which are capable of capturing details not amenable to human eye recognition [76]. Using this radiomic strategy, we further demonstrated that VC affecting different arterial segments could result from specific VC risk traits, such as the degree of advanced age and CKD [77]. Besides, the association between VC and other CKD associated complications, especially low bone volume and impaired bone quality, also varies between studies. Among hemodialysis patients, London and colleagues found that higher VC severities correlated with low bone turnover and osteoporosis [78], whereas Coen et al. disclosed that cardiac and coronary calcification extent did not independently correlate with trabecular bone mass or turnover [79]. This suggests that CKD-associated VC heterogeneity manifests itself in risk factor profiles as well.

VC heterogeneity further implies future risk variations among the affected patients. A multicenter study showed that coronary calcification significantly increased the risk of all-cause mortality among middle-aged or older adults, whereas the risk associated with thoracic aortic calcification was significant only when the severity was moderate or higher [80]. In contrast, another meta-analysis revealed that the presence of coronary and carotid calcifications was significantly associated with a higher risk of kidney stones, whereas abdominal aortic calcification was not [81].

### 4.2 Diagnostic and treatment consideration based on the concept “VC heterogeneity”

The diagnostic approach and screening strategy for VC may be improved by addressing VC heterogeneity. Histopathological and traditional radiographic modalities are ideal approaches for the diagnosis and stratification of VC. Radiomics can be an important means of automatically detecting VC and identifying the origin and phenotype of VC heterogeneity [82]. According to a previous radiomic study, if we aim to screen for VC in patients with CKD instead of in the general population, more attention should be paid to the cephalic-oriented aortic branches and ascending aortae [77]. Novel approaches for VC diagnosis and stratification, such as ultrasound-based approaches, can improve the prediction of peripheral artery disease in patients with diabetes, if appropriately designed to capture segmental heterogeneity [83]. Another useful calcification tendency assessment tool, the T50 test (calciprotein particle maturation time), may also be influenced by the VC heterogeneity phenomenon. A recent study disclosed that vitamin K supplementation in patients with kidney transplant ameliorated arterial stiffness, a surrogate of calcification severity, but failed to alter serum calcification propensity [84].

Treatment directed towards VC will undoubtedly be influenced by VC heterogeneity. As previously explained, pathogenetic heterogeneity, including tissue layer and cellular, subcellular, molecular, and physicochemical variations, serves as the cornerstone of VC heterogeneity. Pharmacological approaches that focus monotonically on any single aspect of each dimension will not suffice to retard VC progression. Pure antioxidants predicted by well-intentioned algorithms to be effective against VC may fail to work after validation [20]. Statins have been shown to exhibit anti-inflammatory, antioxidative, and antithrombotic effects and confer cardiovascular benefits. However, a meta-analysis of 29 observational studies and 12 randomized trials concluded that statin use did not significantly attenuate coronary calcification [85], while another suggested that statins may aggravate coronary calcification [86]. Elevated inorganic phosphate levels have been shown to induce experimental VC [48, 87]; however, phosphate reduction does not consistently reduce VC severity in at-risk populations [88]. These historical examples illustrate failed attempts to pursue a homogenous treatment strategy for VC and point toward a more rational approach by advancing our understanding of VC heterogeneity. Recently, newer generations of VC-ameliorating small molecules have been discovered and tested in clinical trials, especially SNF472, a selective inhibitor of calcium phosphate crystal formation [89]. Therefore, the concept of VC heterogeneity may greatly benefit subsequent efforts to identify potentially effective VC therapeutics. A personalized treatment design will also need to be grounded partially on the concept of VC heterogeneity.

## 5. Conclusion and future perspective

VC is a common finding in advanced aged patients with CKD and diabetes mellitus. Although once perceived as an innocent presentation, VC is now regarded as being equivalent to subclinical vasculopathy, the presence of which significantly worsens long-term clinical outcomes. The recognition of VC can be simple and straightforward, as plain radiography is a convenient way to identify VC. However, many features of VC are not amenable to clear delineation using conventional diagnostic approaches, and its pathophysiology consists of intersecting pathways and networks of cellular, subcellular, and molecular crosstalk. Some of these pathological processes are synergistic and mutually antagonistic, leading to the complex landscape observed in clinical and experimental studies. Collectively, these phenomena can be termed VC heterogeneity and need to be further characterized to uncover effective treatments for this adverse pathology. Clinical implications for VC heterogeneity have begun to be appreciated, involving intra- and inter-individual calcification feature differences, the lack of effectiveness among the tested compounds, and strategies. Indeed, more detailed VC assessment methods are required to optimize care if we aim for individualized management. Longer term studies with interventional design are needed for result validation. We expect that advancements in this field will inspire the development of more rational VC-targeted therapeutics.
